# Supplementary material for: The implications of APOBEC3-mediated C-to-U RNA editing for human disease
Source: Commun Biol. 2024 May 4;7:529. doi: 10.1038/s42003-024-06239-w (PMC11069577; doi:10.1038/s42003-024-06239-w)
Supplement: Supplementary file 2 — Reporting Summary [file 42003_2024_6239_MOESM2_ESM.pdf]

nature portfolio

Corresponding author(s): *Double-anonymous peer review submissions: write DAPR and your manuscript number here instead of author names.*

Last updated by author(s): *YYYY-MM-DD*

Reporting Summary

Nature Portfolio wishes to improve the reproducibility of the work that we publish. This form provides structure for consistency and transparency in reporting. For further information on Nature Portfolio policies, see our Editorial Policies and the Editorial Policy Checklist.

Statistics

For all statistical analyses, confirm that the following items are present in the figure legend, table legend, main text, or Methods section.

n/a	Confirmed	
☐	☒	The exact sample size (*n*) for each experimental group/condition, given as a discrete number and unit of measurement
☒	☐	A statement on whether measurements were taken from distinct samples or whether the same sample was measured repeatedly
☒	☐	The statistical test(s) used AND whether they are one- or two-sided<br>*Only common tests should be described solely by name; describe more complex techniques in the Methods section.*
☒	☐	A description of all covariates tested
☒	☐	A description of any assumptions or corrections, such as tests of normality and adjustment for multiple comparisons
☒	☐	A full description of the statistical parameters including central tendency (e.g. means) or other basic estimates (e.g. regression coefficient) AND variation (e.g. standard deviation) or associated estimates of uncertainty (e.g. confidence intervals)
☒	☐	For null hypothesis testing, the test statistic (e.g. *F*, *t*, *r*) with confidence intervals, effect sizes, degrees of freedom and *P* value noted<br>*Give P values as exact values whenever suitable.*
☒	☐	For Bayesian analysis, information on the choice of priors and Markov chain Monte Carlo settings
☒	☐	For hierarchical and complex designs, identification of the appropriate level for tests and full reporting of outcomes
☒	☐	Estimates of effect sizes (e.g. Cohen's *d*, Pearson's *r*), indicating how they were calculated

Our web collection on statistics for biologists contains articles on many of the points above.

Software and code

Policy information about availability of computer code

Data collection	Coding sequence files were automatically found and downloaded using a custom function which is available via the RNAsee Github (get_cds.py). ClinVar data was downloaded as a single file, so no code was used.
Data analysis	RNAsee v2 was created and used to predict the probability of APOBEC3A/G mediated RNA editing at specific sites. This code is available via the RNAsee Github.

For manuscripts utilizing custom algorithms or software that are central to the research but not yet described in published literature, software must be made available to editors and reviewers. We strongly encourage code deposition in a community repository (e.g. GitHub). See the Nature Portfolio guidelines for submitting code & software for further information.

Data

Policy information about availability of data

All manuscripts must include a data availability statement. This statement should provide the following information, where applicable:

- Accession codes, unique identifiers, or web links for publicly available datasets
- A description of any restrictions on data availability
- For clinical datasets or third party data, please ensure that the statement adheres to our policy

The data used in this work are publicly available. The RNA editing sites used to train and assess RNAsee were originally collated in Asaoka et al, and they may be accessed therein.9 Information on known SNPs were collected from the ClinVar database, which be accessed via https://www.ncbi.nlm.nih.gov/clinvar/. RNA coding

sequences were sourced from the CCDS website, which may be accessed via https://www.ncbi.nlm.nih.gov/projects/CCDS. The CCDS files and ClinVar data used in this paper, along with the scores assigned to each site by RNAsee, have also been made available via http://compbio.buffalo.edu/data/mc_rnasee_biodiv/.

Research involving human participants, their data, or biological material

Policy information about studies with human participants or human data. See also policy information about sex, gender (identity/presentation), and sexual orientation and race, ethnicity and racism.

Reporting on sex and gender	Human subjects not used
Reporting on race, ethnicity, or other socially relevant groupings	Human subjects not used
Population characteristics	Human subjects not used
Recruitment	Human subjects not used
Ethics oversight	Human subjects not used

Note that full information on the approval of the study protocol must also be provided in the manuscript.

Field-specific reporting

Please select the one below that is the best fit for your research. If you are not sure, read the appropriate sections before making your selection.

☒ Life sciences ☐ Behavioural & social sciences ☐ Ecological, evolutionary & environmental sciences

For a reference copy of the document with all sections, see nature.com/documents/nr-reporting-summary-flat.pdf

Life sciences study design

All studies must disclose on these points even when the disclosure is negative.

Sample size	For benchmarking, the largest sample size currently possible (the set of known APOBEC3A/G editing sites) was chosen. For the human health study, the largest sample of polymorphisms available from ClinVar which matched our inclusion criteria was used.
Data exclusions	Sites from the Asaoka et al dataset (benchmarking) or the ClinVar set (assessment of effects on health) were excluded from the primary analysis if the polymorphism did not result in a C>U change in RNA, were not exonic, were synonymous, if an associated coding sequence file could not be found, or if the coding sequence file found did not correspond with the reported polymorphism. Some of these exclusions were required for the feasibility of running RNAsee, (coding sequence exists, C>U change represented), some to ensure the reliability of the data used (coding sequence corresponds with reported polymorphism), and some to ensure that the stated purpose of examining the effects of RNA editing on protein diversity was fulfilled (site is exonic and non-synonymous). These exclusions were consistently and simultaneously applied to all polymorphisms examined.
Replication	Although benchmarking was completed to measure the reliability of the code used in this study, no replication was attempted. The code used is publicly available for reproducibility.
Randomization	For the training of the random forest model and benchmarking of RNAsee, a stratified set of 3086 editing sites, 6172 non-editing sites, and 3086 non-editing sites that scored highly on the rules-based algorithm was created. This set was randomly split 7:3 into the training and testing set. An additional proportional testing set was created by randomly selecting 423806 additional non-editing sites to be added to the testing set so that the positive:negative ratio resembled the original 1:468 prevalence of editing:non-editing sites in the source dataset. No randomization of the ClinVar set occurred.
Blinding	When the subjective judgment of the investigator was necessary during the MeSH term assignment, blinding of the sites associated with each term was carried out. For the benchmarking of RNAsee and the assessment of editing likelihood of ClinVar sites, as the investigator did not have subjective control of the code's output, explicit blinding was not undertaken.

Reporting for specific materials, systems and methods

We require information from authors about some types of materials, experimental systems and methods used in many studies. Here, indicate whether each material, system or method listed is relevant to your study. If you are not sure if a list item applies to your research, read the appropriate section before selecting a response.

Materials & experimental systems

n/a	Involved in the study
☒	☐ Antibodies
☒	☐ Eukaryotic cell lines
☒	☐ Palaeontology and archaeology
☒	☐ Animals and other organisms
☒	☐ Clinical data
☒	☐ Dual use research of concern
☒	☐ Plants

Methods

n/a	Involved in the study
☒	☐ ChIP-seq
☒	☐ Flow cytometry
☒	☐ MRI-based neuroimaging
